# Sleep patterns in patients treated for non-secreting intra- and parasellar tumors: A self-report case-control study

**DOI:** 10.3389/fendo.2022.1044197

**Published:** 2022-11-24

**Authors:** Carl Mathis Wild, Mareike Stieg, Günter K. Stalla, Caroline Jung-Sievers, Matthias K. Auer, Anastasia P. Athanasoulia-Kaspar

**Affiliations:** ^1^ Klinik für Frauenheilkunde und Geburtshilfe, Universitätsklinikum Augsburg, Augsburg, Germany; ^2^ Medicover Neuroendocrinology, Orleansplatz, Munich, Germany; ^3^ Medizinische Klinik und Poliklinik IV, Klinikum der Universität München, Ludwig Maximilian University of Munich, Munich, Germany; ^4^ Chair of Public Health and Health Services Research Institute for Medical Information Processing, Biometry and Epidemiology (IBE) of the Ludwig Maximilian University of Munich, Pettenkofer School of Public Health, Munich, Germany

**Keywords:** pituitary tumors, non-functioning pituitary adenoma, sleep pattern, sleep quality, chronotype, adrenal insufficiency, hydrocortisone

## Abstract

**Purpose:**

In this study we evaluate sleep patterns of patients treated for non-secreting intra- and parasellar tumors and age- and sex-matched healthy controls.

**Methods:**

We conducted a self-report cross-sectional case-control study with 104 patients treated for non-secreting intra- and parasellar tumors and 1800 healthy controls in an 1:8 matching. All subjects answered the Munich ChronoType Questionnaire, whereas patients were provided the Pittsburgh Sleep Quality Index, the Epworth Sleepiness Scale, the Short-Form 36 Health survey, the Beck Depression Inventory and the State-Trait Anxiety Inventory additionally.

**Results:**

Patients treated for non-secreting intra- and parasellar tumors go to bed earlier, fall asleep earlier, need less time to prepare to sleep but also to get up. Additionally, they lie and sleep longer. The subgroup analysis showed that patients with secondary adrenal insufficiency compared to controls experienced shorter daily light exposure and longer sleep latency. Higher hydrocortisone dose (>20mg) was associated with worse score in global, physical and mental health, shorter time to prepare to sleep, earlier sleep onset and longer sleep duration.

**Conclusion:**

Our study shows that patients treated for non-secreting intra- and parasellar tumors, even if successfully treated, experience altered sleep patterns compared to controls. We suggest that managing clinicians should enlighten these possible sleep alterations to their patients and use specific questionnaires to document sleep disturbances. Additionally, when treating patients surgically, especially by transcranial approach, damaging the suprachiasmatic nucleus should be avoided. Furthermore, circadian hydrocortisone replacement therapy ideally with dual-release hydrocortisone - if possible, in a dose not more than 20mg daily - that resembles physiological cortisol levels more closely may be beneficial and could improve sleep patterns and sleep-related quality of life.

## 1 Introduction

Patients with all kinds of pituitary adenomas show disturbed sleep quality (SQ) and sleep patterns (SP) (1). Polysomnographic examination showed that patients with pituitary adenomas have less daytime activity (DA), more fragmented night sleep and less rapid-eye movement and a longer sleep duration (SD) than healthy controls (2, 3). Furthermore, patients report a stronger daytime sleepiness and morning tiredness (4) which may be explained by compression of the suprachiasmatic nucleus of the hypothalamus (SCN) which plays a crucial role in the circadian rhythm (5). Apart from that, changes in the different hormonal axes, especially the hypothalamic-pituitary-adrenal axis, influence the circadian rhythm. It is also well known that apart from hormonal and neural signals, chronotype (CT) differs by age, sex and selected population (6). A chronotype is the behavioral manifestation of underlying circadian rhythms of physical processes and is closely related to circadian rhythm, which controls the day-to-day sleep-wake cycle and release of melatonin in response to environmental cues such as light and temperature (7). There are also great differences between SP on work and free days. Daytime activity and SP on work days are determined by work and most people have to get up earlier contrary to their circadian clock. This leads to sleep deprivation (8).

Replicating the physiological cortisol secretion is key in the treatment of glucocorticoid insufficient individuals and optimization may enhance the quality of life. However, this is not always the case, even if adequate hydrocortisone substitution is being administered, both with conventional or dual-release hydrocortisone. It is well known, that glucocorticoids are powerful circadian entrainers of peripheral clocks and the immune system is one of the most sensitive to this daily ‘reset’ (9). Patients with adrenal insufficiency report of increased fatigue and decreased quality of life (10) but also significant alterations in circadian gene expression compared to healthy controls (11).

In the present study, we want to examine if there is a significant difference in terms of SP between patients treated for non-secreting intra- and parasellar tumors (NST) near the SCN and a healthy control group (CG). Furthermore, we want to assess SP in terms of the presence of secondary adrenal insufficiency (SAI) and patients without SAI and secondarily to explore the impact of disease and treatment characteristics such as daily hydrocortisone (HC) dose.

In a further step, we want to test if health-related quality of life (HRQoL) is affected by SP and which factors affect SQ.

## 2 Materials and methods

### 2.1 Subjects

One hundred four patients with NST from the Endocrine Outpatient Clinic of Max Planck Institute of Psychiatry in Munich were recruited between 2013 and 2014 and included in our study. Patients were either provided with a set of questionnaires during a routine visit to our department or contacted by mail within a twelve-month window after their last visit. Visual field defects (VFD) were diagnosed by standard Goldmann perimetry. We excluded 7 shift workers due to disturbance of normal chronobiology. Patients who used an alarm clock on free days (n=18) were also excluded.

Subjects of the CG were matched 1:8 for age and sex before dropout and recruited by the WeP, a platform coordinated by Prof. T. Roenneberg, head of human chronobiology at the Institute of Medical Psychology of the Ludwig Maximilian University of Munich. Controls were healthy volunteers and data were collected between 2001 and 2014 in Germany. Healthy volunteers could register and answer an online questionnaire and were evaluated only by the Munich ChronoType questionnaire (MCTQ). Further available information about the controls were age, sex and BMI. The study was approved by the medical ethics committee of the Ludwig Maximilian University of Munich and was conducted within the framework of NeoExNET. All patients and controls gave their written informed consent.

### 2.2 Diagnosis of pituitary insufficiencies in the patient group

Pituitary function and adequateness of treatment were evaluated by basal measurements of thyroid-stimulating hormone (TSH), free thyroxine (FT4), free triiodothyronine (FT3), insulin-like growth factor 1 (IGF-1), luteinizing hormone (LH), follicle-stimulating hormone (FSH), total testosterone (in men) or estradiol (in women) and cortisol.

In women, in the fertile age range, gonadotropin deficiency was defined as low LH and FSH in women with secondary amenorrhea for at least one year. In postmenopausal women, gonadotropin deficiency was documented when gonadotropin levels were inappropriately low for the post- menopausal age, whereas in men it was defined as a low testosterone level with an inappropriate low LH and FSH. In women treatment included estrogen/progesterone replacement or in the case of past hysterectomy only estrogen. Most women were receiving transdermal estrogen preparations. In men, gonadotropin deficiency was substituted with testosterone as gel or intramuscular injection. Thyrotropin deficiency was defined as low FT4 with an inappropriately low serum TSH and was treated with levothyroxine. SAI was documented by an inadequate low basal cortisol at 8:00 am and an inadequate rise of cortisol after corticotropin-stimulation test or insulin hypoglycemia test (IHT). Patients with SAI were treated with conventional HC usually divided into 2-3 doses per day or by dual-release formulation (Plenadren ^®^). GHD was suspected when baseline fasting IGF-1 was lower than the normal values for the age- and sex- range. If this was the case, the patient, depending on his/her comorbidities and health status underwent a GHRH/arginine or an IHT test. The peak value of growth hormone which was considered abnormal and defined a GHD was in the GHRH/arginine test for normal weight persons (BMI < 25 kg/m2) < 11 mcg/l, for overweight patients (BMI 25 - 30 kg/m2) < 8 mcg/l and for obese patients (BMI > 30 kg/m2) < 4 mcg/l. In the case of IHT the peak GH value of 3 mcg/l was used to define GHD. GH replacement therapy was offered to all patients with documented GHD when no contraindications were present. Diabetes insipidus was diagnosed after a water deprivation test and was treated with desmopressin.

### 2.3 Questionnaires

We used a self-constructed questionnaire to collect personal data and medical history. Data of the medical history were also collected from the patient’s medical record. Furthermore, standard generic questionnaires listed below were used to assess parameters such as SQ, SP, CT, HRQoL, depression and anxiety. Patients got the self-constructed questionnaire, SF-36, BDI, STAI, PSQI, ESS and MCTQ. Subjects of the control group only got the MCQT.

### 2.4 Quality of life questionnaires

The Short-Form 36 Health survey (12) (SF-36) is a patient-reported questionnaire with 36 items. Patients used its German form, that has been validated as a valuable tool in epidemiological and clinical studies (13). It is a common tool to survey patient health and is also often used to survey quality of life. It is a multi-item scale that assesses one global score, two sum scores for mental and physical health and eight subscales: (1) vitality; (2) physical functioning; (3) bodily pain; (4) general health perception; (5) physical role functioning; (6) emotional role functioning; (7) social role functioning; (8) mental health. The score of each scale reaches from 0-100 with lower scores showing a higher grade of impairment and higher scores showing a lower grade of disability.

The Beck Depression Inventory (14, 15) (BDI) is a patient-related survey with 21 questions to assess the severity of depression. Patients were administered its German version, that demonstrates good reliability and validity in clinical and nonclinical samples (14). Each question refers to the feeling in the last week and has four possible answers ranging in intensity. The score is divided in four categories: (1) no/minimal depression (0-13); (2) mild depression (14-19); (3) moderate depression (20-28); (4) severe depression.

The State-Trait Anxiety Inventory (16, 17) (STAI) is a patient-related measure of trait and anxiety. There are 20 items for each scale. Every item consists of a short statement that the patient has to rate on a four-point scale. For anxiety and trait, the score ranges from 20 and 80. Higher scores on the anxiety set of items suggest anxiety and worry. Higher scores on the trait scale suggest a higher level of sadness and self-deprecation. The German Version that patients filled in has been validated (18).

### 2.5 Sleep questionnaires

#### 2.5.1 Pittsburgh Sleep Quality Index

The Pittsburgh Sleep Quality Index (19) (PSQI) is a 19- item self-report questionnaire to assess sleep quality over a 1-month interval. Each question has an interval score from 0-3 with higher scores indicating a greater impairment of sleep quality. The 19 questions are computed to seven components: (1) subjective sleep quality, (2) sleep latency, (3) sleep duration, (4) habitual sleep efficiency, (5) sleep disturbances, (6) use of sleeping medication, and (7) daytime dysfunction. The sum of the scores of the seven components results in a global sleep-quality index with higher scores indicating a higher degree of sleep disturbance. Participants filled in a German adaptation (20) of the Pittsburgh Sleep Quality Index.

#### 2.5.2 Epworth Sleepiness Scale

The Epworth Sleepiness Scale (ESS) is a short self-report questionnaire to assess the probability of falling asleep during the daytime (21, 22). Patients rate their chance to fall asleep in eight different situations on a scale from 0 to 3 with higher scores showing a higher degree of sleepiness. A total score above 10 is defined as elevated. Patients have been administered the German version of ESS that has been validated for application in German-speaking populations (23).

#### 2.5.3 Munich ChronoType questionnaire

The Munich ChronoType questionnaire (MCTQ) is a self-rated scale to assess primary sleep times, such as bed- and rise-times, plus the clock time of becoming fully awake as well as sleep latency and inertia. It consists of 17 items addressing questions about bedtime (BT), time preparing to sleep (TSP), sleep latency (SL), sleep end (SE), time to stand up (SI), light exposure (LE) and alarm clock separated for free (f) and work (w) days. In this study, we used data only from free days, as we know that there are great differences between SP on work and free days (24). Based on these facts, values like sleep onset (SO=TSP+SL), time to get up (GU= SE + SI), sleep duration (SD=SE-SO) and total bedtime (TBT=GU-BT) are computed. The estimation of chronotype is based on the midpoint between SO and SE on free days without the use of an alarm clock (MS=(SE-SO)/2) (8, 24, 25). Its original form is in German and the questionnaire has been validated (8).

#### 2.5.4 Statistical analysis

All statistical analyses were performed with a statistical package for the Social Science 25.0 Software (SPSS 25.0). Demographic data are presented by means and standard deviations and frequencies. For differences in categorical variables between groups, we used the Chi-square test. Two- sided Student’s t-test was used for normally distributed metric variables and Mann–Whitney U Test for not normally distributed variables. Significance was set at the 0.05 level.

In a first step, we compared all patients with the CG in terms of SP. In a second step we compared patients with and without SAI in terms of SP, SQ and HRQoL. We also conducted a subgroup analysis comparing patients with SAI with CG and patients without SAI and CG in terms of SP. For multiple comparison we used the false discovery rate (26). We conducted eleven analyses so that the corrected significance level is p=0.0273.

In an exploratory analysis, we focused on the correlation between different potentially influential variables and the SF-36 global- mental- and physical health score and SP. For variables with normal distribution, we used Pearson’s correlation. For variables without normal distribution or linear correlation we used Spearman’s correlation.

## 3 Results

### 3.1 Patients and controls

We received questionnaires from 133 patients with NST. We excluded 7 shift workers due to disturbance of normal chronobiology. Patients who used an alarm clock on free days (NST: n = 18; CG: n = 31) and 4 patients with missing data were also excluded so that in the end data from 104 (57 men and 47 women). Eighty-two of them had been diagnosed with non-functioning pituitary adenomas (NFPA), eight patients with craniopharyngioma, six patients with meningioma, two patients with Rathke cleft cyst, two patients with granuloma of the pituitary gland, one patient with a colloid cyst, one patient with a gangliom, one patient with a chondroma clivus and one patient with NFPA and meningioma. Eighty-eight (87.1%) had been treated at least once by primary transsphenoidal surgery and 24 (23.1%) with additional radiotherapy and were considered to be cured.

The arithmetic mean age for the patients was 63.7 ± 13.9 years. Mean BMI was 26.7 ± 4.2 kg/m2. All of them were on stable replacement therapy for hypopituitarism, except for growth-hormone deficiency (GHD) with was untreated in 59.6% of GHD patients, mainly due to patients’ wish. Fifty-four (51.9%) patients suffered from SAI and were under HC substitution with a mean daily HC-dose of 18.9 ± 4.9 mg, whereas four of them were currently receiving dual-release HC (Plenadren^®^). The control group of the WeP consisted of 1800 patients with an average age of 57.3 ± 14.6 years, divided into 918 men and 882 women with a BMI of 25.7 ± 4.4 kg/cm2. The distribution of age and sex was similar between patients and the control group. Further baseline and disease characteristics are presented in **Table 1**.

**Table 1:** Baseline and disease characteristics of patients and controls.

	Patients (n=104)			Controls (n = 1800)		
	N (%)	Mean	sd	N (%)	Mean	sd
Age (years)	n.a.	63.7	13.9	n.a.	57.3	14.6
Gender (male)	57 (54.8)	n.a.	n.a.	918 (51)	n.a.	n.a.
Body mass index (kg/m2)	n.a.	26.7	4.2	n.a.	25.7	4.4
Transsphenoidal surgery	88 (87.1)	n.a.				
Radiotherapy	24 (23.1)	n.a.				
Time since diagnosis	n.a.	13.4	8.0			
Visual field defects (VFD)	24 (23.1)	n.a.	n.a.			
Intact pituitary function	18 (17.3)	n.a.	n.a.			
Secondary adrenal insufficiency(SAI)	54 (51.9)	n.a.	n.a.			
Under replacement	54 (100)	n.a.	n.a.			
Growth hormone deficiency	72 (69.2)	n.a.	n.a.			
Under replacement	42 (58.3)	n.a.	n.a.			
Gonadotropin deficiency	74 (71.2)	n.a.	n.a.			
Under replacement	37 (50)	n.a.	n.a.			
Thyrotropin deficiency	60 (57.7)	n.a.	n.a.			
Under replacement therapy	60 (57.7)	n.a.	n.a.			
Diabetes insipidus	12 (11.9)	n.a.	n.a.			

n.a., non-applicable.

### 3.2 Sleep patterns

#### 3.2.1 Comparison with controls

In a first step, we compared sleep patterns with the MCTQ between patients and controls. We could show that patients had significantly earlier bet time and sleep onset, shorter time prepare to sleep and time to stand up but significantly longer total bed time and sleep duration. There were no significant differences regarding chronotype (MS) (**Table 2**).

**Table 2:** Sleep patterns in patients and controls.

	Patients		Controls		
	Mean	sd	Mean	sd	p
MCTQ					
BT *	22:53:38	01:08:36	23:44:17	01:18:23	**<0.001**
TSP *	23:18:27	01:10:20	23:55:46	01:18:54	**<0.001**
SL**	00:18:25	00:20:31	00:14:58	00:16:06	0.099
SO *	23:37:13	01:13:46	00:10:17	01:18:05	**<0.001**
SE *	07:54:57	01:03:18	07:42:38	01:36:58	0.069
SI **	00:07:53	00:07:58	00:19:49	00:23:21	**<0.001**
GU *	08:03:41	01:05:32	08:04:44	01:35:53	0.880
TBT **	09:10:17	01:05:23	08:21:01	01:19:59	**<0.001**
SD **	08:20:40	01:08:13	07:32:31	01:21:46	**<0.001**
MS *	03:47:15	00:59:21	03:58:25	01:25:31	0.101
LE **	03:03:43	01:59:33	03:33:11	02:05:55	0.038

Data are presented as mean with standard deviation or as percentages.

*clock time in h:min

**duration in h:min

Munich ChronoType questionnaire (MCTQ), Bed time (BT), Time prepare to sleep (TSP), Sleep latency (SL), Sleep onset (SO), Sleep end (SE), Time to stand up (SI), Time to get up (GU), Total bed time (TBT), Sleep duration (SD), Chronotype (MS - midpoint between SO and SE on free days), Light exposure (LE). Bold values denote statistical significance at the p < 0.0273 level ( corrected significance level).

#### 3.2.2 Subgroup analysis in the patient group

In a further step we conducted a subgroup analysis in the patient group comparing SP in patients with to patients without SAI. Apart from shorter light exposure in patients with SAI (SAI: 02:33 ± 01:36 h; without SAI: 03:33 ± 02:13 h; p=0.022) and a trend to longer sleep latency (SAI: 0:22 ± 0:22 h; without SAI: 0:14 ± 0:18 h; p= 0.044) there was no significant difference in sleep patterns between patients with and without SAI (data not shown). Additionally, we compared patients with and without SAI to the CG. In contrast to patients without SAI, patients with SAI experienced shorter LE (SAI: 02:33 ± 01:36 h CG: 03:33 ± 02:06 h; p=0.003) and longer SL (SAI: 0:22 ± 0:22; CG: 0:15 ± 0:16 h; p=0.018) compared to the controls. All other data are similar to the comparison of all patients to the controls(data not shown).

In terms of HRQoL as measured by BDI, STAI, SF-36 and in terms of SQ/daytime sleepiness as measured by PSQI and ESS respectively we could not find any significant difference between patients with or without SAI in the standardized questionnaires (data not shown).

#### 3.2.3 Correlation analysis in the patient group

In a further step we focused on the correlation between different sleep parameters and HRQoL. HRQoL measured by the three sum-scores of the SF-36 was negatively correlated with SQ measured by PSQI, with the daytime sleepiness measured by ESS and SL (measured by MCTQ). LE was positively correlated with SF-36 mental health sum score. No other parameters of the MCTQ were correlated with SF-36 (**Table 3**).

**Table 3:** Correlation analysis of sleep parameters and sleep quality with SF-36.

	SF-36 global health		SF-36 physical health		SF-36 mental health	
	cc	p	cc	p	cc	p
MCTQ						
BT	0.098	0.385	0.102	0.363	0.153	0.555
TSP	0.089	0.479	0.108	0.386	0.042	0.724
SL	**-0.303**	**0.006**	**-0.300**	**0.006**	**-0.314**	**0.003**
SO	-0.012	0.922	0.006	0.962	-0.064	0.585
SE	0.017	0.877	0.008	0.940	-0.001	0.991
SI	-0.017	0.881	0.051	0.652	-0.105	0.332
GU	0.030	0.789	0.027	0.808	0.001	0.996
TBT	-0.105	0.356	-0.116	0.305	-0.089	0.415
SD	0.079	0.523	0.054	0.662	0.103	0.383
MS	0.028	0.821	0.040	0.744	-0.030	0.803
LE	0.165	0.182	0.125	0.310	**0.264**	**0.024**
**PSQI**	**-0.492**	**<0.001**	**-0.463**	**<0.001**	**-0.479**	**<0.001**
**ESS**	**-0.238**	**0.028**	-0.167	0.125	**-0.291**	**0.005**

For the abbreviations, please refer to **Table 2**. Bold values denote statistical significance at the p < 0.05 level.

Furthermore, we examined whether age, gender, BMI, disease or treatment characteristics such as VFD (dichotomous -yes/no), SAI (dichotomous - yes/no), operation (dichotomous - yes/no), radiation (dichotomous - yes/no) and high HC dose (>20mg) (dichotomous - yes/no) correlate with SP and SQ (measured by MCTQ and PSQI) and daytime sleepiness (ESS).

Neither operation nor radiation or VFD were correlated with SP or SQ. Age was negatively correlated with BT (cc: -0.351, p<0.001), TSP (cc: -0.370; p=0.001), SO (cc: -0.318, p=0.003), SE (cc: -0.526, p<0.001), SI (cc: -0.415, p<0.001), GU (cc: -0.554, p<0.001) and MS (cc: -0.489, p<0.001). Female gender was positively correlated with SL (cc: 0.325, p=0.001), SI (cc: 0.380, p<0.001) and PSQI (cc: 0.286, p=0.004) and negatively correlated with LE (cc: -0.285, p=0.010). SL was positively correlated with PSQI (cc: 0.520, p < 0.001) and negatively with LE (cc: -0.361; p = 0.001). LE was negatively correlated with the presence of SAI (cc: -0.253; p=0.022) and PSQI (cc: -0.385, p<0.001) but not with impairment of the other pituitary axes or other disease characteristics (data not shown). In a further step, we examined whether patients with high HC dose (>20mg daily) experienced altered HRQoL or SP. The cutoff of 20mg HC daily has been chosen, as it is well known that this is the standard substitution dose and that a higher dose is rarely necessary (21, 22). We could find that a high HC dose was negatively correlated with SF-36 global (cc: -0.425; p=0.004), physical (cc: -0.382; p=0.009) and mental health (cc: -0.347; p=0.016). In terms of sleep parameters, high HC dose was correlated with earlier TSP (cc:_-0.341; p=0.022), earlier SO (cc: -.369; p=0.012) and longer SD (cc: 0.413; p=0.004). No correlation with SQ or CT could be stablished (data not shown).

## 4 Discussion

The aim of this study was to assess sleep parameters in patients with NST with a particular focus on the role of SAI and HC substitution in regard to SP. Our study shows that patients with NST, even if successfully treated, experience altered SP compared to matched controls. NST patients go to bed and fall asleep earlier, need less time to prepare to sleep and also get up earlier. Additionally, they seem to lie and sleep longer. Regarding CT, we could not establish any significant difference between patients and controls.

The subgroup analysis focusing on patients with SAI could show that patients with SAI compared to non-SAI patients experienced shorter daily LE and longer SL and a shorter daily LE compared to controls. The correlation analysis in the patient group revealed that no disease or treatment characteristics were correlated with sleep parameters apart from the presence of SAI, which was found to be negatively correlated with LE. We could find that a high HC dose is associated with worse SF-36 global, physical and mental health. In terms of sleep parameters, a high HC dose was correlated with shorter TSP, earlier SO and longer SD.

Numerous studies have reported that pituitary adenoma patients suffer from fatigue, daytime somnolence, depression and anxiety, symptoms which often persist even after adenoma resection and despite adequate hormonal replacement (27–29). Our findings indicating that NST patients go to bed and fall asleep earlier could be explained by the fact that NST patients experience significantly more fatigue and daytime sleepiness than controls, and these altered SP seem to be an attempt to compensate for this daytime fatigue and somnolence. Our results showing that NST patients stay longer in bed and sleep longer are in accordance with the findings of Joustra et al., which showed that patients with NFPA spent more time in bed (longer TBT). Data from studies in the same patient group suggest that patients with NST suffer from SCN dysfunction either due to mechanical damage due to compression by the tumor or due to its surgical treatment (3). Dysfunctional SCN is being correlated among others with increased total sleep time, a finding that could be confirmed in our study as increased sleep duration was found in our patient group. Indirectly, our results indicate a dysfunctional SCN in patients with NST.

However, van der Klaauw et al. (4, 30) could not establish any difference between NFPA and craniopharyngioma patients and controls in terms of SP. It is worth mentioning, however, that in both studies only few parameters of MCTQ have been evaluated (SD, SO, MS) and data from a self-selected small control group were analyzed. Furthermore, we examined these parameters in a larger patient and control group. In a similar study by Biermasz (2) patients reported a higher number of sleep disturbances such as longer sleep latency, early awakening, breathing difficulties or uncomfortable temperature compared to controls. However, also in this study, a 1:1 matching of self-selected controls was used.

Another aim of our study was to correlate disease and treatment characteristics with the observed altered SP. SP were found to be correlated only with the presence of SAI, which was negatively correlated with daily LE and positively with SL compared to patients with intact corticotropic axis but also controls. This observation could not be explained by a difference in depression or quality of life or anxiety scores, as no such difference could be established. This observation is in accordance with the results of our previous study, implying that patients with SAI did not have an impaired quality of life (10).

As in the study of Joustra et al. the presence of treated hypopituitarism, in general, was not associated with any altered sleep parameters (3). However, when SAI was separated and analyzed for its individual influence, treated patients with SAI indeed experienced altered SP with increased intradaily variability and decreased intradaily stability (3). As similar changes were also found in patients with NFPA and intact adrenal axis, Joustra studied patients with primary adrenal insufficiency (Addison’s disease – AD), which experience adrenal insufficiency without the effect of the pituitary mass. It could be shown that AD patients report normal daytime sleepiness and subjective sleep quality, parameters that were profoundly altered in NFPA patients (3). This indicates that decreased daytime functioning reflects inadequate daytime energy levels, results that support our observations showing that patients with SAI experience lower daily LE. It is well known that increased physical activity improves sleep and mood outcomes in inactive people with insomnia (3). Patients with AD, where the pituitary mass effect is missing and only the influence of hydrocortisone substitution is present, report similar impairments in terms of fatigue and general QoL as NFPA patients. However, it seems that subjective SQ and daytime sleepiness are not affected, while both were profoundly altered in NFPA patients. This is supported by the results in AD patients reported by Løvås et al. (31), who did not identify specific sleep disturbances characteristic for patients with AD rather than increased daytime fatigue without more daytime sleepiness than normal. In our study SL and LE correlated with SF-36 mental health and they were negatively correlated to each other. Patients with AD, when provided with continuous subcutaneous hydrocortisone infusion, experienced an improvement in subjective vitality and QoL (32). It is therefore plausible that treating patients with SAI with replacement regimes such as dual-release hydrocortisone that provides a more circadian-based serum cortisol profile (33) could lead to decreased daytime fatigue, increased vitality and more LE, leading to decreased sleep latency. Another possible explanation for longer SL could be the supraphysiological evening levels of cortisol related to the evening hydrocortisone dose, an observation that was also made by Joustra et al. (3). Furthermore, melatonin reduces sleep-onset latency and it is well documented that patients with NST including craniopharyngiomas and NFPA experience a disturbed melatonin rhythm (34). The melatonin effect could partially explain the longer SL.

According to this hypothesis, patients undergoing multiple daily dose replacement therapy for adrenal insufficiency show significant alterations in circadian gene expression compared to healthy controls (9, 11).

We can, however, still not answer the question regarding causality and our results allow two explanations: One possible explanation of longer SL in patients with SAI apart from reduced LE could be the intake of an evening hydrocortisone dose, which might lead to supraphysiological evening levels of cortisol and therefore longer SL. Alternatively, due to the documented increased fatigue, they might take a nap in the course of the day, a fact that could lead to shorter daily LE and longer SL at night. In contrast to AD patients, our results may not only reflect the intrinsic imperfections of the thrice daily oral hydrocortisone substitution but seem to be rather a combination of the dysfunction of the SCN and disturbed melatonin rhythm.

In a previous study of our group, it could be shown that patients on a higher substitution dose with HC experienced an impaired HRQoL measured by SF-36 global and physical health score (8). When having a closer look at the subgroup of patients with SAI in this study, data suggest that LE and SL were both negatively correlated with the presence of SAI. Regarding substitution dose, it seems that taking more than 20mg hydrocortisone daily is associated with a shorter time to prepare to sleep, earlier sleep onset and longer sleep duration. Even if thinking that taking more HC daily leads to increased energy level, our study shows that this is associated with sleep alterations that rather indicate more fatigue. One explanation for this could be, that patients with high HC dose experience more fatigue and try to compensate for this by increasing the HC dose. Our results are in accordance with previous studies, which showed, that there is an optimum to be attained since patients on higher doses of nocturnal glucocorticoid frequently report sleep disturbance (35), results that are also known from the research in Cushing syndrome (36).

In terms of HRQoL, as measured by BDI, STAI and SF-36 and in terms of SQ/daytime sleepiness as measured by PSQI and ESS respectively, we could not find any significant difference between patients with or without SAI in the standardized questionnaires. In a further step, our results indicate that HRQoL was negatively correlated with SQ, daytime sleepiness and SL, a result which is in accordance with the study of van der Klaauw (4). Being more exposed to daily light as measured by LE was positively correlated with SF-36 mental health sum score.

The following limitations of our study should be acknowledged. In the control group, only the MCTQ was available and not other questionnaires such as ESS and PSQI. Additionally, the accuracy of such questionnaires depends on self-reporting and self-report studies have validity issues. Patients may exaggerate symptoms in order to make their situation seem worse, or they may under-report the severity or frequency of symptoms in order to minimize their problems. Patients might also simply be mistaken or misremember the material covered by the survey. Furthermore, most of our patients received conventional hydrocortisone and only a few dual-release hydrocortisone. Also, the strong correlation between SL and SF-36 mental health and subjective sleep quality has to be controlled by objective SP.

In conclusion, patients with NST seem to experience altered sleep patterns compared to controls. Furthermore, patients with SAI report longer sleep latency and shorter daily light exposure. We suggest that managing clinicians treating patients with NST should enlighten these possible sleep alterations on their patients and could use specific questionnaires in the daily routine to document sleep disturbances. Additionally, when treating NST patients surgically, one should try to avoid damaging the SCN, especially when treating with the transcranial approach. Furthermore, circadian hydrocortisone replacement therapy ideally with dual-release hydrocortisone - if possible, in a dose not more than 20mg daily - that resembles physiological cortisol levels more closely may be beneficial and could improve sleep patterns and sleep-related quality of life.

## Data availability statement

The original contributions presented in the study are included in the article/supplementary material. Further inquiries can be directed to the corresponding author.

## Ethics statement

The studies involving human participants were reviewed and approved by the medical ethics committee of the Ludwig Maximilian University of Munich. The patients/participants provided their written informed consent to participate in this study.

## Author contributions

CW and MA conceived the presented idea. CW developed the theory and performed the analytic calculations and the numerical simulations. CJ-S, MS, MA, and GS verified the analytical methods. AA-K supervised the project and took the lead in writing the manuscript. All authors provided critical feedback and helped shape the research, analysis, and manuscript.

## Acknowledgments

We would like to thank Prof. Dr. Till Roenneberg, Head of Human Chronobiology in the Institute of Medical Psychology of the Ludwig Maximilian University of Munich for providing the control group.

## Conflict of interest

CW, MA, CJ-S declare no conflict of interest. MS received speakers’ fees by Novartis, Ipsen, Pfizer, consultancy fees by Novartis and research funding by Novartis, Pfizer. AA-K and GS received consultancy fees and/or reimbursements of delegate fees for conferences/educational events and/or travel expenses and/or funding for research projects from Pfizer, Ipsen, Lilly, Shire, Novartis, Sandoz, NovoNordisk, HRA and Recordati.

## Publisher’s note

All claims expressed in this article are solely those of the authors and do not necessarily represent those of their affiliated organizations, or those of the publisher, the editors and the reviewers. Any product that may be evaluated in this article, or claim that may be made by its manufacturer, is not guaranteed or endorsed by the publisher.

## Abbreviations

SQ, Sleep Quality; SP, Sleep Patterns; DA, Daytime activity; SCN, Suprachiasmatic nucleus of the hypothalamus; CT, Chronotype patterns; SAI, Secondary adrenal insufficiency; NST, Non-secreting intra- and parasellar tumors; NFPA, Non-functioning pituitary adenomas; CG, Control group; IHT, Insulin hypoglycemia test; HC, Hydrocortisone; GHD, Growth-hormone deficiency; GH, Growth hormone; BDI, Beck Depression Inventory; STAI, State-Trait Anxiety Inventory; PSQI, Pittsburgh Sleep Quality Index; ESS, Epworth Sleepiness Scale; SF-36, Short-Form 36 Health survey; MCTQ, Munich ChronoType questionnaire; BT, Bed time; TSP, Time prepare to sleep; SL, Sleep latency; SE, Sleep end; SI, Time to stand up; LE, Light exposure; SO, Sleep onset; GU, Time to get up; SD, Sleep duration; TBT, Total bed time; MS - midpoint between SO and SE on free days, Chronotype; AD, Addison’s disease
